# Discussing overweight in dogs during a regular consultation in general practice in the Netherlands

**DOI:** 10.1111/jpn.13558

**Published:** 2021-06-18

**Authors:** Celine M. Aldewereld, Evelyn M. Monninkhof, Floor M. Kroese, Denise T. D. de Ridder, Mirjam Nielen, Ronald J. Corbee

**Affiliations:** ^1^ Department of Clinical Sciences Faculty of Veterinary Medicine Utrecht University Utrecht The Netherlands; ^2^ Julius Center for Health Sciences and Primary Care University Medical Center Utrecht Utrecht University Utrecht The Netherlands; ^3^ Social, Health and Organizational Psychology Utrecht University Utrecht The Netherlands; ^4^ Department of Population Health Sciences Faculty of veterinary medicine Utrecht University Utrecht The Netherlands

**Keywords:** barriers, canine, communication, motivation, obesity

## Abstract

In previous studies, it has been demonstrated that, similar to general practitioners, veterinarians find it difficult to discuss overweight in dogs. This study aimed to provide insight in the barriers and motivators for veterinarians to discuss overweight in dogs and to compare the results with findings from human medicine. Sub‐hypotheses were postulated based on existing literature to investigate if lack of time, fear of offending clients, or lack of skills were potential barriers, and if feeling responsible and feeling compassion were potential motivators for veterinarians to discuss overweight in dogs. To this end, an online survey (*n* = 59) was conducted. Furthermore, 15 small animal clinicians working in general practice were interviewed by semi‐structured face‐to‐face interviews. Results from the online survey indicated that veterinarians find it sometimes difficult to discuss overweight in dogs. Veterinarians who responded to the online survey did not experience strong barriers but did make use of motivators (e.g. feeling responsible and feeling compassion) when discussing overweight in dogs. Interestingly, results from the semi‐structured face‐to‐face interviews showed that the responding veterinarians did experience strong barriers, as well as motivators, when discussing overweight in dogs with their clients. The most prominent barrier was customer dissatisfaction, whereas lack of time and lack of skills were also experienced. The most prominent motivator was feeling responsible for animal health and preventive veterinary medicine. These findings were strikingly similar to previous research on discussing childhood overweight by general practitioners. To improve treatment and prevention of overweight in dogs, veterinarians need more communication skills and should be more aware of the motivators that drive their self‐motivation. Improving awareness on overweight and its comorbidities should be a One Health issue.

## 1 INTRODUCTION

Overweight has a high prevalence within the canine population with recently published percentages varying between 56–59% (Pet obesity prevention, 2020; Read, 2019). Overweight and obesity are defined as abnormal or excessive fat accumulation that presents a risk for the dog's health. A dog is considered to be overweight when the current bodyweight is more than 10–20% above the optimal bodyweight, and obese when the current bodyweight is more than 20–30% above the optimal bodyweight (German, 2016). Determining if a dog is overweight or obese can be difficult, as many different dog breeds exist, and also within breeds there are large variations in optimal bodyweights. Therefore, the use of bodyweight alone is usually not a sensitive marker for overweight. A body condition score (BCS) has been developed to give insight in the body condition of dogs (Laflamme, 1997). Dogs with a BCS over 5 out of 9 are considered overweight, whereas dogs with a BCS over 6 out of 9 are considered obese (German, 2016). Similar to overweight people, dogs that are chronically overweight have an increased risk of metabolic, cardiovascular, respiratory, urogenital and orthopaedic disorders, and overweight is associated with a decreased life expectancy (Chandler et al., 2017). Therefore, it is important to recognize overweight early, to improve health care and to prevent the development of obesity or other comorbidities related to overweight. This should be a One Health approach, as overweight dogs often have overweight owners (Nijland et al., 2010). Furthermore, a complex relationship commonly exists between owners and their companion animal, particularly around feeding behaviour. Obese companion animals commonly live alongside caregivers who are also struggling with their own bodyweight (Candellone et al., 2017). An important owner‐related risk factor for the development of obesity or other comorbidities related to overweight in dogs is the ignorance and/or unawareness of dog owners regarding overweight. Dog owners often do not recognize that their dog is overweight. Previous research showed that 65% of surveyed pet owners misjudged the BCS of their dog, where underestimation of the body condition was most common (Eastland‐Jones et al., 2014; Holmes et al., 2007). Owner disagreement was found to be especially common in veterinarian‐defined overweight dogs (White et al., 2011). When overweight is discussed with pet owners, only 19% disagreement has been reported (Cairns‐Haylor and Fordyce, 2017). This stresses the importance of good client education, the need for veterinarians to determine and communicate the presence and level of overweight, and to discuss overweight with their clients. Veterinarians have indicated that overweight is currently not getting the attention, it deserves within their profession (Yeates and Main, 2011) as it is often missed by pet owners (Belshaw et al., 2018), or not addressed as an issue by pet owners (Cairns‐Haylor and Fordyce, 2017). Veterinarians should not only point towards owners but should also take their own responsibility. Despite the essential role of veterinarians regarding health care and prevention of diseases, it appears that veterinarians have difficulty admitting or discussing overweight of their clients' dogs (Cairns‐Haylor and Fordyce, 2017; Kipperman and German, 2018). This is also reflected by the fact that veterinarians seldom record the overweight status of dogs in their patient files (Rolph et al., 2014). Difficulty discussing overweight is not only a concern among veterinarians. General practitioners also addressed experiencing barriers when discussing overweight with parents of overweight children (Gerards et al., 2012; Van der Maas et al., 2020). This study aimed to provide more insight in the barriers and motivators for veterinarians to discuss overweight in dogs and to compare the results with findings from human medicine.

## 2 MATERIAL AND METHODS

To provide insight in the barriers and motivators for veterinarians to discuss overweight in dogs, an online survey (quantitative analysis) and semi‐structured face‐to‐face interviews (qualitative analysis) were conducted.

### 2.1 Quantitative analysis

For the online survey, we aimed at a minimum of 50 respondents who were working as companion animal clinician in general practice within the Netherlands, which was logistically feasible and deemed representative for this pilot study. The veterinarians were recruited by the use of social media. The link to the survey was posted in a closed Facebook group for veterinarians, called ‘Het Dierenartsen Gilde’, on the Website of Utrecht University, and on the website of the Royal Dutch Veterinary Association (KNMvD). The survey was administered online using Qualtrics software (2017). The survey consisted of 26 statements and responses were given on 1–5 Likert scales. The survey started with four questions about how and when veterinarians discuss a dog's overweight during general consultation (e.g. ‘I use the BCS chart to discuss overweight’). Respondents specified their level of agreement with the statements by choosing from the following answer categories: 1 = never, 2 = rarely, 3 = sometimes, 4 = often, 5 = always. After that, 5 variables were assessed reflecting possible barriers and motivators: lack of time, fear of offending clients, and lack of skills; feeling responsible and feeling compassion. These statements had to be answered using a 1–5 Likert scale (i.e. 1 = totally disagree 2 = disagree 3 = neutral 4 = agree 5 = totally agree). The complete survey, along with reliability statistics of the 5 variable scales, is shown in Appendix 1.

#### 2.1.1 Data analysis of the quantitative analysis

The results collected from the online survey were analysed using SPSS Statistics for Windows 24.0. Only data sets with more than 80% of the survey questions answered were analysed, to avoid including data sets with too little data for analysis, and to exclude participants that decided to withdraw during the survey. Results are presented as Likert scales for all categories, and a Cronbach's alpha was calculated for each category of barriers and motivators to measure internal consistency. A Cronbach's alpha of over 0.6 was regarded as satisfactory, and a Cronbach's alpha of over 0.7 as good reliability.

### 2.2 Qualitative analysis

#### 2.2.1 Respondents

For the semi‐structured face‐to‐face interviews, we aimed to include 15 small animal clinicians currently working in general practices in the Netherlands, similar to the human pilot study (van der Maas et al., 2020) which was deemed representative to elucidate the most important themes. To recruit veterinarians, we spread an advertisement by using social media (self‐selection strategy) and actively contacted randomly selected practices (from the list of practices obtained from the KNMvD) by telephone. The advertisement was posted in the Facebook group ‘Het Dierenartsen Gilde’, on the Website of Utrecht University, and on the KNMvD website. All in‐depth interviews were conducted by one researcher (CA) at a location of the clinician's preference (e.g. the general practice or at their home). The interviewer was a veterinary student in her final Master year. The interviews focussed on personal experiences that veterinarians had encountered in their practice when discussing overweight in dogs, with specific emphasis on barriers and motivators. The main topics of the interviews were based on the barriers mentioned in previous research and were divided into the following subcategories: lack of time, fear of offending clients, and lack of skills; feeling responsible, and feeling compassion. The interviews consisted of pre‐specified open questions to motivate the respondents to speak freely during the conversation. The interviewer used the same questions in each conversation, as shown in Appendix 2, to ensure that the questions in subsequent interviews were not adapted to previous experiences of the interviewer. However, if something was unclear, the interviewer asked the respondents to elaborate, or apply prewritten follow‐up questions. Preferably questions started with: What…?, How…?, Why…?, etc. For example; ‘What problems do you experience while discussing overweight of a dog with its owner’? The interviewer made notes during the interviews and a member check was performed by giving a summary of the identified barriers followed by confirmation of the veterinarian. If the veterinarian did not experience barriers, the interviewer catechized certain barriers found in the literature at the end of the interview, and ask whether these barriers played a role for the respondent. The interview ended by showing the researcher's appreciation for participation. During the inclusion period, weekly sessions (CA, EM and RC) were scheduled to evaluate the interview progress and to discuss difficulties. The interviews were recorded by a smartphone with the app Recorder 5.0.0.1 (when permission was given by the respondents). The interviews were subsequently coded by using QSR NVivo10.2. The interviews were analysed after the inclusion period by using a modified version of the constant comparative method to extract different themes and to develop codes (thematic analysis) similar to van der Maas et al., 2020. Coding was performed by labelling all parts of the interview that were relevant to the research question (CA). Thereafter, sessions of peer debriefing and peer‐reviewing with one other researcher (EM, clinical epidemiologist) was performed. Inconsistencies were resolved by discussion (EM and CA). After the peer‐reviewing, a final analysis of the data was performed by evaluating the themes once again. A modified version of the coding outline was developed (CA). Eventually, the coding outline was discussed with all authors. Statistical analysis was not performed for the qualitative study, as is standard for qualitative studies with the aim to demonstrate different experiences (Belshaw et al., 2018).

## 3 RESULTS

### 3.1 Quantitative research

**FIGURE 1:**
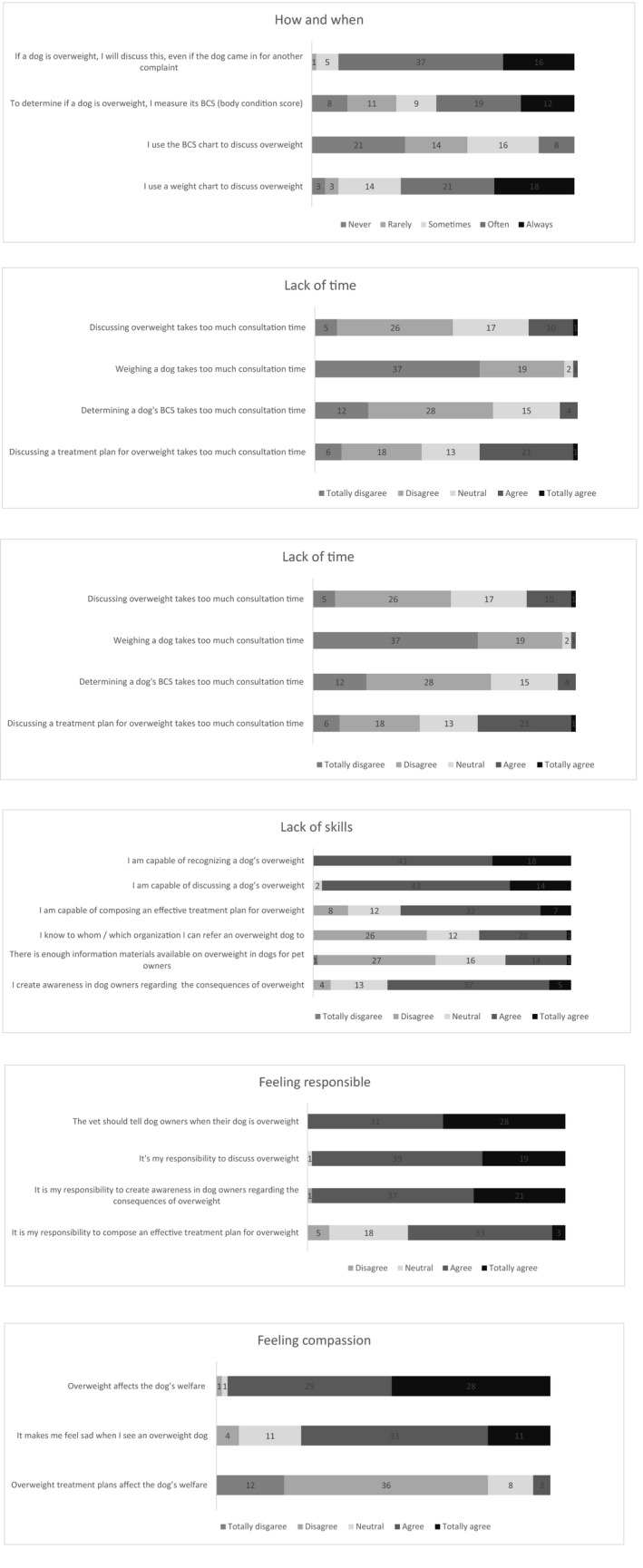
Results of the online survey. (i) General questions‐How and when: Mean score (SD) per item 4.2 (0.6), 3.3 (1.3), 2.2 (1.1), 3.9 (1.1). (ii) Barriers‐Lack of time: Cronbach's alpha was 0.677, and was 0.736 if the second question is taken out. Mean score per item 2.6 (0.9), 1.4 (0.7), 2.2 (0.8), 2.9 (1.1). Mean barrier score 2.3 (1.0), and 2.6 (1.0) if the second question was taken out. (iii) Fear of offending clients: Cronbach's alpha was 0.093, and is 0.716 if the first question was taken out. Answers for questions 3 and 4 were inverted for Cronbach's alpha calculation and to calculate the mean barrier score. Mean score per item 3.8 (0.9), 1.9 (0.8), 2.6 (1.0), 2.9 (0.8), 1.8 (0.9). Mean barrier score 2.8 (1.2), and 2.6 (1.1) if the first question was taken out. (iv) Lack of skills: Cronbach's alpha was 0.610, and is 0.698 if the fifth question was taken out. Mean score per item 4.3 (0.5), 4.2 (0.5), 3.6 (0.9), 2.9 (0.9), 2.8 (0.9), 3.7 (0.7). To calculate the mean barrier score the results were inverted. Mean barrier score 2.4 (0.9), and 2.2 (0.9) if the fifth question was taken out. (v) Motivators‐Feeling responsible: Cronbach's alpha was 0.706, and 0.802 if the final question was taken out. Mean score per item 4.5 (0.5), 4.3 (0.5), 4.3 (0.6), 3.6 (0.7). Mean motivator score 4.2 (0.7), and 4.4 (0.5) if the final question was taken out. (vi) Feeling Compassion: Cronbach's alpha was 0.170, and 0.622 if the final question was taken out. Mean score per item 4.4 (0.6), 3.9 (0.8), 2.0 (0.7). Mean motivator score 3.4 (1.3), and 4.1 (0.8) if the final question was taken out. Data set from 59 respondents of an online survey among veterinarians regarding overweight in dogs, using a 5‐point Likert scale (never, rarely, sometimes, often, always for the general questions, and totally disagree, disagree, neutral, agree, totally agree for each category of barriers and motivators). The 1–5 scale was used to compute the mean score (±SD) per item

Seventy‐one respondents filled in the online survey. Data of two respondents were excluded because less than 80% of the survey questions were answered, and data of 10 respondents were excluded because these veterinarians were working outside the Netherlands, leaving a complete data set from 59 respondents for further analysis (all 59 respondents answered all the questions of the questionnaire). Ninety‐three percent of the respondents (*n* = 55) were female and mean age was 35 years (SD = 10.5), so female veterinarians, and younger veterinarians were over‐represented. The results of the online survey are demonstrated in Figure 1.

### 3.2 General questions: How and when

The majority of the respondents (90%, *n* = 53) indicated to always or often discuss overweight, even if the dog came in for an unrelated complaint. About half of the respondents (54%, *n* = 32) claimed to always or often use the BCS (i.e. during every consultation), whereas 13% (*n* = 8) of the respondents claimed to use the BCS to discuss overweight with their clients. A total of 65% (*n* = 38) of the respondents agreed to always or often use a weight chart to discuss overweight.

### 3.3 Barriers

#### 3.3.1 Lack of time

Lack of time was not considered a barrier (mean barrier score = 2.6, SD = 1.0). Cronbach's alpha was 0.677 for the questionnaire, and 0.736 if the question about weighing dogs is left out of the questionnaire (which can be considered as general practice and therefore was expected not to be a barrier). More than half of the respondents (52%, *n* = 31) disagreed or totally disagreed that discussing overweight takes up too much consultation time, whereas discussing a treatment plan was considered the most important barrier (agree or totally agree 37%, *n* = 22).

#### 3.3.2 Fear of offending clients

Fear of offending clients was not considered a barrier (mean barrier score = 2.6, SD = 1.1). Cronbach's alpha was 0.093, and 0.716 if question about finding it difficult to tell owners that their dog is overweight is left out of the questionnaire (as the majority of the respondents (77%, *n* = 45) found it difficult to tell dog owners that their dog is overweight, but did not seem to experience the other barriers in this subgroup to the same extent). Strikingly, 90% (*n* = 53) of the respondents disagreed or totally disagreed with the statement: ‘I will not discuss overweight because I am afraid of negative reactions’. When dealing with an overweight owner, 61% (*n* = 36) of the respondents disagreed or totally disagreed with this statement. Only five percent of the respondents (*n* = 3) indicated fear of losing clients as a barrier.

#### 3.3.3 Lack of skills

Lack of skills was not considered a barrier (mean barrier score = 2.2, SD = 0.9). Cronbach's alpha was 0.610, and 0.698, if the question about availability of materials is left out of the questionnaire (which is not directly related to skill, but rather an indicator of awareness about tools that are available to assist in effectively discussing overweight). The respondents regarded themselves competent in recognizing and in discussing a dog's overweight (100% (*n* = 59) and 97% (*n* = 57), respectively). Sixty‐three percent of the respondents (*n* = 37) regarded themselves competent in setting up an effective treatment plan. Thirty‐five percent of the respondents (*n* = 21) indicated to know to which organization they can refer an overweight dog.

### 3.4 Motivators

#### 3.4.1 Feeling responsible

Feeling responsible was considered a motivator (mean motivator score = 4.4, SD = 0.5). Cronbach's alpha was 0.706, and 0.802 if the question about designing a treatment plan is left out of the questionnaire (as this might be done by another member of the veterinary health care team, e.g. vet nurses). Almost all respondents agreed that veterinarians should play a role in signalling (100%, *n* = 59) and discussing (98%, *n* = 58) overweight in dogs. Sixty‐one percent of the respondents (*n* = 36) agreed that it is the responsibility of the veterinarian to give dog owners insight in the severity of their dog's overweight.

#### 3.4.2 Feeling compassion

Feeling compassion was considered a motivator (mean motivator score = 4.1, SD = 0.8). Cronbach's alpha was 0.170, and 0.622 if the question about the effect of a treatment plan on welfare is left out of the questionnaire. Only 2 respondents (3%) did not agree with the statement that overweight affects a dog's welfare. The majority of the respondents agreed with the statement ‘It makes me feel sad when a dog is overweight’ (76%, *n* = 45). Specific comments and notes of respondents that they wanted to share in the final part of the survey are shown in Appendix 3.

##### Qualitative research

Sixteen respondents were recruited, 13 by social media, and three by random selection by phone calls. One of the respondents was not currently working in a companion animal practice at the moment and, therefore, excluded from the study, leaving 15 data sets for analysis. Forty percent of the respondents were male (*n* = 6), and 60% female (*n* = 9), their mean age was 41 years (SD = 10.7). The duration of the interviews ranged from 26 min to 66 min (Mean = 42 min, SD = 11.3). The themes and subthemes extracted from the interviews are presented in Table 1. Most respondents (*n* = 12, 80%) indicated that the prevalence of overweight in dogs within their practice was about 50% (range 12–75%). Fourteen of the 15 respondents (93%) indicated using BCS to determine if a dog is overweight. In addition to BCS, some respondents (*n* = 3, 20%) mentioned to use the weight progression scale offered by numerous veterinary practice software packages. Ten of the fifteen respondents (67%) stated that it is the responsibility of veterinarians to discuss overweight. These ten respondents also mentioned that it is their task to create awareness among the dog owners regarding their dog's body condition, and the possible consequences related to overweight, since it is experienced that owners often do not recognize their dog as overweight, as was mentioned by respondent#5; ‘*When I see an overweight dog, I must tell the owner,* *as a lot of owners are totally unaware of it’*. Most respondents (*n* = 11, 73%) stated that discussing overweight and creating awareness about overweight with pet owners is an important aspect of preventative veterinary medicine. A great majority of the respondents (*n* = 14, 93%) indicated to feel responsible for the animal, as the animal cannot speak for itself, for example, respondent#4 indicated; ‘*It's my job to address it for the animal since the animal cannot address the issue himself*’. When asking about barriers they experience when discussing overweight, numerous respondents (*n* = 9, 60%) mentioned they had none. However, when the interview progressed different barriers were identified, finally resulting in all the respondents admitting to have experienced barriers when discussing overweight. All respondents mentioned owner resistance as a barrier, which is illustrated by a quote of respondent#4; ‘*Well, if an owner expresses obvious resistance,* *then that is the end of the conversation*’. Owner resistance seemed to be based on two different aspects, that is, defensive behaviour and indifference. Nearly all the respondents (*n* = 14, 93%) mentioned that owners immediately started to defend themselves when the subject overweight was introduced. Owners often blame other people or address the fact that the dog only eats little amounts of food, so they are not responsible and/or cannot do anything about it, which happened in respondent#14's consultation room; *‘People are always blaming someone else, for example The husband says: “Yes, my wife always gives him treats”, and then next time the wife comes in and says: “Yes,* *my husband always gives him treats”’*. All 15 respondents mentioned that owner's resistance is sometimes caused by indifference and that owners simply lack interest or do not seem to be concerned about their dog's overweight. Owners will state that the dog being overweight is simply part of its life, or appoint that their overweight dog is doing fine and, therefore, doesn't need treatment. Some respondents (*n* = 4, 27%) mentioned owners who appointed to find an overweight dog more attractive because overweight dogs look ‘healthier’ or ‘happier’. Respondent#4 mentioned; *‘One of the situations is that people truly do not agree with the fact that their dog is overweight and simply deny it: "No, he's just fine!" or “He likes the good life (just like us)!" or "He's not bothered by it at all!", Yes, you can try to start up a discussion then, but that's* *just no use…’* Lack of motivation was mentioned by almost all respondents (*n* = 14, 93%). Lack of motivation refers to unmotivated owners who do not act upon their dog's overweight in the course of time. Lack of motivation was a frustrating barrier for most respondents (*n* = 13, 87%), which had also led to demotivation of themselves. Respondent#12 demonstrated this clearly; *‘If an owner says: “He has always weighed the same” I try to indicate that it would be better for the dog to lose weight. But, if you know you already had the same conversation with the same owners a year before,* *I become less motivated to address the issue again’*. About half of the respondents mentioned fear of losing clients as a barrier. Some respondents mentioned to experience this, especially when encountering a new client. The respondents mentioned to be careful discussing overweight, or sometimes deliberately not discuss overweight, for that particular reason. Some respondents mentioned to experience a barrier when repeatedly having to address overweight with owners that are reluctant to act on their dog's overweight. They mentioned to discuss overweight carefully because they do not want to upset the owners with the possible result of losing them and finally losing control over the patient, as is exemplified by respondent#10; ‘*You want to keep some control over the dog's wellbeing. Especially if it is a regular client,* *it is very frustrating if the client leaves you for another vet due to discussions about overweight’*. Twelve of the fifteen respondents (80%) experienced an overweight owner as a barrier. Some respondents mentioned to feel uncomfortable with the situation and thus started discussing overweight in a more general way. Respondent#1 confessed; *‘I notice by myself that I bring up the issue a bit more general, or use less firm expressions, for example, I tell them: “Yes, he is a bit too heavy.” When in fact he is seriously obese, and then it is often ignored by the owner*. *Maybe that is due to the fact that we do not feel comfortable to address the issue as firm as we should’*. Time was experienced as a barrier by 10 of the 15 respondents (67%) when discussing overweight. If there is a certain time pressure, respondents mentioned not to discuss overweight. Most respondents indicated to make a note in the patient file about the dog being overweight in these situations, leaving the discussion for a next consultation. A smaller number of respondents indicated a lack of skills as a barrier. Some respondents found it difficult to apply BCS correctly and did not know at which BCS they should start the conversation. Some respondents mentioned not being capable of motivating owners and did not know how to deal with resistant owners, like respondent#1, who mentioned; *‘Sometimes you think: I mentioned it last year, and I mentioned it again, and they just do not listen, so you simply do not address it anymore. And that might also be a barrier I think; how to explain the seriousness of the problem,* *which is a real challenge’*.

**TABLE 1:** Results of the semi‐structured face‐to‐face interviews

Subgroup and factors	Mentioned
How and when	
Use of BCS to determine if a dog is overweight	14
Use of BCS to discuss overweight	8
Discussion of overweight by wet nurse	5
Use of body weight to discuss overweight	13
Discusses health benefits and risks of overweight	12
Discusses overweight every consultation	4
Discusses overweight at annual health check	10
Records body weight and body condition each consultation	12
Lack of time	10
Fear of offending clients	15
Afraid of customer loss	7
Overweight owner	12
Resistant owner	15
Indifference of the owner	15
Defensive owner	14
Owner sees vet as salesman of pet food	6
Lack of skills	8
Vet does not have enough knowledge about overweight	7
Vet does not have enough knowledge about nutrition	6
Feeling responsible	
Creating awareness and discussion about overweight is my responsibility	11
I am responsible for discussing overweight as it is part of preventative veterinary medicine	10
Feeling compassion	7
Others	
Unawareness of their dog's overweight by the owner	15
Lack of motivation by the vet, as the vet had negative experiences in the past	7
Frustration by the vet	7
Unmotivated owner	13
Owners do not want to stop giving treats	7
Owners do not want to feed less food	4
Owners do not want to switch diet	4
Financial constraints of the owner prevents proper treatment plan	9
Family situation of the owner prevents proper treatment plan	7
Lack of follow‐up	9
Lack of support for the owner by family, friends	2
Dog is always hungry during weight loss program	4

Note

Data of 15 semi‐structured face‐to‐face interviews with veterinarians regarding overweight in dogs. The answers to the questions were divided in subgroups and factors and the numbers of respondents that mentioned this subgroup and/or factor are given.

## 4 DISCUSSION

Overweight is recognized as a health issue by all of the veterinary respondents in this study, and about half of them make use of BCS on a regular basis, which is in agreement with previous studies (Vandendriessche et al., 2017, Yeates and Main, 2011). Providing tools, such as BCS charts and instruction videos, and making veterinarians aware of these tools may improve their use (Bergler et al., 2016; WSAVA global nutrition guidelines, 2020). The estimation of the prevalence of overweight in Dutch small animal practices (50%) is in agreement with recent studies in the USA and in the UK 56–59% (Pet obesity prevention, 2020; Read, 2019). The wide variation between the self‐reported perceptions of the prevalence of overweight by veterinarians can be just a matter of differences in perception. It might also partly be explained by regional differences, which is similar to a study in the United Kingdom, where the prevalence of overweight was lower in the London area compared to rural regions (White et al., 2011), although this was not tested in our study. Veterinarians experience barriers and motivators when discussing overweight in dogs during a regular consultation in general practice. Interestingly, the barriers were more prominent in the semi‐structured face‐to‐face interviews, compared to the online survey, and even in the semi‐structured face‐to‐face interviews, the barriers were only mentioned a bit later in the interview. Veterinarians are probably not aware of these barriers at first but do admit to experience these barriers when asked more specifically. In the quantitative study, all the barrier categories had a mean score below 3, indicating that, on average, the respondents did not indicate to experience these barriers when discussing overweight in dogs. The respondents in the quantitative study did indicate that they find it difficult to tell pet owners that their dog is overweight, but other the barriers within the category ‘fear of offending clients’ were not experienced by them (mean score 2.6 ± 1.1). Customer dissatisfaction and fear for negative reactions seemed to be the strongest barriers for the responding veterinarians in the qualitative study, which is similar to studies in both veterinary medicine (Cairns‐Haylor and Fordyce, 2017; White et al., 2011) and human medicine (Gerards et al., 2012; Van der Maas et al., 2020). However, only a small part of the population (11% of the general Dutch population) indicates finding it inappropriate for general practitioners to address a patient's overweight status at a general consultation not related to overweight (Reitsma and De Jong, 2010). The percentage of dog owners finding it inappropriate when their veterinarian discusses overweight is currently unknown. They do however report that if the veterinarian did not raise the issue, they assumed it was not abnormal or concerning (Belshaw et al., 2018). As mentioned above, a positive association exists between the Body Mass Index (BMI) of pet owners and the BCS of their dogs. This increases the likelihood that the overweight dog has an overweight owner, which could result in an increased fear for the veterinarian of offending their clients, resulting into customer dissatisfaction (Cairns‐Haylor and Fordyce, 2017; Nijland et al., 2010). This was mentioned as a barrier by some of the respondents in both the quantitative and the qualitative study. Lack of time was mentioned as a barrier by some of the respondents, which is similar to previous studies (Belshaw et al., 2018; Cairns‐Haylor and Fordyce, 2017). To overcome this, veterinary nurses can take over part of the veterinarian's tasks, however, it is still important that the veterinarian should start the conversation or provides support to the veterinary nurse as was indicated by several respondents (Appendix 3). Some respondents have mentioned deliberately not to discuss overweight when they have little time. These findings are in accordance with earlier findings in veterinary medicine (Cairns‐Haylor and Fordyce, 2017) and human medicine (Gerards et al., 2012). Lack of skills is also mentioned as a barrier by some of the respondents, which is in agreement with the finding that some youth healthcare professionals indicated improvement is possible related to their skills, for example, communication skills (Gerards et al., 2012). As mentioned above many pet owners do not recognize their pet as overweight (Holmes et al., 2007), and sometimes they regard overweight pets as more attractive as was mentioned in this study, which is in agreement with findings in show dogs (Corbee, 2013). Owners also lack knowledge on proper feeding and exercise (Webb et al., 2018). Interpreting such findings in terms of psychological models of behaviour, one could say that owners' *attitudes* and *perceived behavioural control* tend to be low, which according to the Theory of Planned Behavior (Ajzen, 1985; together with perceived *social norms* about the behaviour) is likely to yield low intentions to change behaviour (i.e. to adapt their dog's eating or exercise patterns). This makes it difficult for veterinarians to discuss overweight. This Theory of Planned Behavior can also be applied to veterinarians: to optimize their intention to discuss overweight with their clients, they should not only consider overweight as an important aspect of preventative health care. They should also be aware that their clients, the public and other veterinarians (significant others) want them to act. Furthermore, veterinarians should experience that this behaviour is easy to perform (high perceived behavioural control). The barriers identified in this study point to possible areas of improvement in that regard. Veterinarians feel insecure about their knowledge on nutrition and weight loss plans, and they have poor confidence about follow‐up questions that pet owners might have (Belshaw et al., 2018; Bergler et al., 2016). Despite the inclusion of nutrition education in veterinary curriculum, many practitioners feel this is insufficient and face challenges in starting evidence‐based discussions with clients (Kamleh et al., 2020). Further nutrition‐related education (and development of communication skills) is needed at undergraduate and post‐graduate level.

Altogether, it is clear that veterinarians require good communication skills and need more knowledge about overweight and its comorbidities to effectively discuss overweight with their clients. A previous study demonstrated that improved communication with pet owners is associated with fewer complaints and higher levels of satisfaction experienced by the owner (Adams and Frankel, 2007). Making sure that pet owners recognize the problem of overweight and the perceived benefits of a weight loss programme is the first step. If veterinarians can effectively support pet owners by providing tools and follow‐up plans to increase success rates, the barrier of perceived behavioural control of both pet owners and veterinarians can be reduced, which will further stimulate veterinarians and pet owners to act (Ajzen, 1985). Positive motivational attitude from the veterinarian will further increase chances of success (Rollnick et al., 2010).

The responding veterinarians in both studies largely agreed that they feel responsible for signalling and discussing overweight in pets (i.e. in the quantitative study the motivator ‘Feeling responsible’ had a mean score of 4.2 ± 0.7 and 10 out of 15 respondents of the qualitative study mentioned their own responsibility as a motivator). This is in agreement with a previous veterinary study (Yeates and Main, 2011) and with human medicine, as previous studies showed that 91% of the general practitioners are convinced that general practitioners should play a role in signalling childhood obesity (Paulis, 2016; Paulis et al., 2012). Feeling compassion was experienced as a motivator for veterinarians to discuss a dog's overweight, as was indicated by a mean score of 4.1±0.8 in the quantitative study. Overall, about 75% of the respondents indicated that it makes them feel sad to see an overweight dog.

### 4.1 Limitations

This study has included 1 data set of 59 respondents from the online survey and 15 data sets from the semi‐structured face‐to‐face interviews in small animal clinicians working in general practice in The Netherlands. They self‐selected themselves for both parts of the study, thus creating sample selection bias. In line with previous studies, male respondents are hard to recruit, and therefore, female veterinarians were over‐represented (Belshaw et al., 2018). The authors expect that male and female veterinarians will have similar barriers and motivators towards discussing overweight, but cannot rule out possible gender bias. Younger veterinarians were over‐represented as they are more likely to participate in questionnaires. The authors expect that older and younger veterinarians will have similar barriers and motivators towards discussing overweight, but cannot rule out possible age bias. The qualitative study included veterinarians that were slightly older. Increased experience can change people's behavioural or control beliefs and might affect intention to act (e.g. previous issues of non‐compliance by pet owners has been mentioned as a barrier) (Ajzen, 2011). Respondents who are more committed to the topic might be more appealed to participate. However, one of the respondents of the qualitative study did mention that his participation was more as a statement against all the fuss around overweight. In accordance, the online survey seemed to have one respondent who seemed less committed to the topic overweight by stating in the comment section: *‘There are more important problems than overweight animals’*, as shown in Appendix 3. Observer error and bias was possible for the qualitative analysis, as only one researcher (CA) executed the interviews and did most part of processing the data. Since qualitative analysis was based on interpretation of the interviews this could have led to a decreased reliability of the study. However, there were two sessions planned with another researcher (EM) to analyse the interview data together, to increase the reliability of coding the interviews. All the interviews were analysed, then the codes were compared and analysed for a second time.

Future studies should focus on evaluating interventions that make veterinarians more confident in discussing overweight with their clients, making use of overcoming barriers and stimulation of motivators. A similar study on barriers and motivators can also be conducted in vet nurses.

## 5 CONCLUSION

Veterinarians do experience barriers as well as motivators when discussing overweight in dogs with their clients. The most prominent barrier was customer dissatisfaction, whereas lack of time and lack of skills were also experienced as barriers. The most prominent motivator was feeling responsible for animal health and preventive veterinary medicine. These findings were strikingly similar to previous research in youth health care professionals and general practitioners. To improve treatment and prevention of overweight in dogs, veterinarians need more communication skills, and veterinarians should be more aware of the motivators that drive their self‐motivation. Improving awareness on overweight and its comorbidities should be addressed as a One Health issue.

## ANIMAL WELFARE STATEMENT

The authors confirm that the ethical policies of the journal, as noted on the journal's author guidelines page, have been adhered to. No ethical approval was required under Dutch legislation. All respondents participated voluntarily and could withdraw from the study at any time.

## Supporting information

Appendix 1

Appendix 2

Appendix 3

## References

[jpn13558-bib-0001] Adams, C. L. , & Frankel, R. M. (2007). It may be a dog's life but the relationship with her owners is also key to her health and well being: Communication in veterinary medicine. Veterinary Clinics of North America: Small Animal Practice, 37, 1–17. 10.1016/j.cvsm.2006.10.003 17162108

[jpn13558-bib-0002] Ajzen, I. (1985). From intentions to actions: A theory of planned behavior. In J. Kuhl , & J. Beckman (Eds.), Action control: From cognition to behavior (pp. 11–39). Springer.

[jpn13558-bib-0003] Ajzen, I. (2011). The theory of planned behaviour: Reactions and reflections. Psychology & Health, 26, 1113–1127. 10.1080/08870446.2011.613995 21929476

[jpn13558-bib-0004] Belshaw, Z. , Robinson, N. J. , Dean, R. S. , & Brennan, M. L. (2018). Owners and veterinary surgeons in the united kingdom disagree about what should happen during a small animal vaccination consultation. Veterinary Science, 5, 7. 10.3390/vetsci5010007 PMC5876577 29346332

[jpn13558-bib-0005] Bergler, R. , Wechsung, S. , Kienzle, E. , Hoff, T. , & Dobenecker, B. (2016). Nutrition consultation in small animal practice ‐ a field for specialized veterinarians. Tierarztl Prax Ausg K Kleintiere Heimtiere, 44, 5–14. 10.15654/TPK-150154 26661505

[jpn13558-bib-0006] Cairns‐Haylor, T. , & Fordyce, P. (2017). Mapping discussion of canine obesity between veterinary surgeons and dog owners: a provisional study. Veterinary Record, 180, 149. 10.1136/vr.103878 27986895

[jpn13558-bib-0007] Candellone, A. , Morgan, D. , Buttignol, S. , & Meineri, G. (2017). Leaner, healthier, happier together–a family‐centred approach to weight loss with the overweight dog and her caregivers. Veterinary Sciences, 22, 41. 10.3390/vetsci4030041 PMC5644662 29056699

[jpn13558-bib-0008] Chandler, M. , Cunningham, S. , Lund, E. M. , Khanna, C. , Naramore, R. , Patel, A. , & Day, M. J. (2017). Obesity and associated comorbidities in people and companion animals: A one health perspective. Journal of Comparative Pathology, 156, 296–309. 10.1016/j.jcpa.2017.03.006 28460795

[jpn13558-bib-0009] Corbee, R. J. (2013). Obesity in show dogs. Journal of Animal Physiology and Animal Nutrition, 97, 904–910. 10.1111/j.1439-0396.2012.01336.x 22882163

[jpn13558-bib-0010] Eastland‐Jones, R. C. , German, A. J. , Holden, S. L. , Biourge, V. , & Pickavance, L. C. (2014). Owner misperception of canine body condition persists despite use of a body condition score chart. Journal of Nutritional Science, 3, e45. 10.1017/jns.2014.25 26101613 PMC4473163

[jpn13558-bib-0011] Gerards, S. M. , Dagnelie, P. C. , Jansen, M. W. , De Vries, N. K. , & Kremers, S. P. (2012). Barriers to successful recruitment of parents of overweight children for an obesity prevention intervention: a qualitative study among youth health care professionals. BMC Family Practice, 13, 37. 10.1186/1471-2296-13-37 22591134 PMC3403855

[jpn13558-bib-0012] German, A. J. (2016). The growing problem of obesity in dogs and cats. Journal of Nutrition, 136, 1940S–1946S. 10.1093/jn/136.7.1940S 16772464

[jpn13558-bib-0013] Holmes, K. , Morris, P. , Abdulla, Z. , Hackett, R. , & Rawlings, J. (2007). Risk factors associated with excess body weight in dogs in the UK. Journal of Animal Physiology and Animal Nutrition, 91, 166–167. 10.1111/j.1439-0396.2007.00680_9.x

[jpn13558-bib-0014] Kamleh, M. K. , Khosa, D. K. , Dewey, C. E. , Verbrugghe, A. , & Stone, E. A. (2020). Ontario veterinary college first‐year veterinary students' perceptions of companion animal nutrition and their own nutrition: Implications for a veterinary nutrition curriculum. Journal of Veterinary Medical Education, 48, e0918113r1. 10.3138/jvme.0918-113r1 32412363

[jpn13558-bib-0015] Kipperman, B. S. , & German, A. J. (2018). The responsibility of veterinarians to address companion animal obesity. Animals, 8, 143. 10.3390/ani8090143 PMC6162666 30134516

[jpn13558-bib-0016] Laflamme, D. P. (1997). Development and validation of a body condition score system for dogs; a clinical tool. Canine Practice, 22, 10–15.

[jpn13558-bib-0017] Nijland, M. L. , Stam, F. , & Seidell, J. C. (2010). Overweight in dogs, but not in cats, is related to overweight in their owners. Public Health Nutrition, 13, 102–106. 10.1017/S136898000999022X 19545467

[jpn13558-bib-0018] Paulis, W. D. (2016). Childhood Obesity in Primary Care: Not yet General Practice. Erasmus University Rotterdam. http://hdl.handle.net/1765/80158/.

[jpn13558-bib-0019] Paulis, W. , de Jong, A. , van der Wouden, H. , Van Avendonk, M. , & Boukes, F. (2012). Kinderen met obesitas in de huisartsenpraktijk. Tijdschrift Voor Gezondheidswetenschappen, 90, 171–175. 10.1007/s12508-012-0068-y

[jpn13558-bib-0020] Pet obesity prevention (2020). Figures on the prevalence of dog overweight 2018. Retrieved from https://petobesityprevention.org/.

[jpn13558-bib-0021] Read, C. (2019). The growth of pet obesity. Veterinary Record, 185, 1–3. 10.1136/vr.l6606 31727730

[jpn13558-bib-0022] Reitsma, M. , & De Jong, J. (2010). Ongevraagd advies bij overgewicht. Huisarts en Wetenschap, 53, 583. 10.1007/s12445-010-0275-1

[jpn13558-bib-0023] Rollnick, S. , Butler, C. C. , Kinnersley, P. , Gregory, J. , & Mash, B. (2010). Motivational interviewing. BMJ, 340, c1900. 10.1136/bmj.c1900 20423957

[jpn13558-bib-0024] Rolph, N. C. , Noble, P.‐J.‐ M. , & German, A. J. (2014). How often do primary care veterinarians record the overweight status of dogs? Journal of Nutritional Science, 3, e58. 10.1017/jns.2014.42 26101626 PMC4473162

[jpn13558-bib-0025] Van der Maas, J. C. , Corbee, R. J. , Kroese, F. M. , De Ridder, D. T. D. , Vos, R. C. , Nielen, M. , & Monninkhof, E. (2020). Discussing overweight in children during a regular consultation in general practice: a qualitative study. BMC Family Practice, 21, 18. 10.1186/s12875-020-1088-3 31992231 PMC6986030

[jpn13558-bib-0026] Vandendriessche, V. L. , Picavet, P. , & Hesta, M. (2017). First detailed nutritional survey in a referral companion animal population. Journal of Animal Physiology and Animal Nutrition, 101, 4–14. 10.1111/jpn.12621 28627059

[jpn13558-bib-0027] Webb, T. L. , Krasuska, M. , Toth, Z. , du Plessis, H. R. , & Colliard, L. (2018). Using research on self‐regulation to understand and tackle the challenges that owners face helping their (overweight) dogs lose weight. Preventive Veterinary Medicine, 159, 227–231. 10.1016/j.prevetmed.2018.08.017 30314787

[jpn13558-bib-0028] White, G. A. , Hobson‐West, P. , Cobb, K. , Craigon, J. , Hammond, R. , & Millar, K. M. (2011). Canine obesity: is there a difference between veterinarian and owner perception? Journal of Small Animal Practice, 52, 622–626. 10.1111/j.1748-5827.2011.01138.x 22017760

[jpn13558-bib-0029] WSAVA global nutrition guidelines (2020). Global Nutrition toolkit. Retrieved from https://wsava.org/global‐guidelines/global‐nutrition‐guidelines/.

[jpn13558-bib-0030] Yeates, J. W. , & Main, D. C. J. (2011). Veterinary surgeons' opinions on dog welfare issues. Journal of Small Animal Practice, 52, 464–468. 10.1111/j.1748-5827.2011.01095.x 21896020

