# Supplementary material for: Discussing overweight in dogs during a regular consultation in general practice in the Netherlands
Source: J Anim Physiol Anim Nutr (Berl). 2021 Jun 18;105(Suppl 1):56–64. doi: 10.1111/jpn.13558 (PMC8519133; doi:10.1111/jpn.13558)
Supplement: Supplementary file 1 — Appendix 1 [file JPN-105-56-s001.docx]

Appendix 1. Online survey

| General questions |
| --- |
| What is your gender?  - Male  - Female |
| What is your age? |
| Are you currently working as a small animal clinician in general practice in The Netherlands? |
| How and when is overweight addressed? |
| Answer categories: never, rarely, sometimes, often, always |
| If a dog is overweight, I will discuss this, even if the dog came in for another complaint |
| To determine if a dog is overweight, I measure its BCS |
| I use the BCS chart to discuss overweight |
| I use a weight chart to discuss overweight |
| Possible barrier: Lack of time |
| Answer categories: Totally disagree – disagree – neutral – agree – totally agree |
| Discussing overweight takes too much consultation time |
| Determining the dog's BCS takes too much consultation time |
| Weighing a dog takes too much consultation time |
| Discussing a treatment plan for overweight takes too much consultation time |
| Possible barrier: Fear of offending clients |
| Answer categories: Totally disagree – disagree – neutral – agree – totally agree |
| I find it difficult to tell dog owners that their dog is overweight |
| I do not discuss overweight because I'm afraid of negative reactions |
| I think clients do not find it a problem, if I discuss their dog’s overweight |
| I do not experience it as a problem to discuss a dog's overweight if the owner is overweight too |
| I'm afraid of customer loss if I discuss overweight during a consultation |
| Possible barrier: Lack of skills |
| Answer categories: Totally disagree – disagree – neutral – agree – totally agree |
| I am capable of recognizing a dog’s overweight |
| I am capable of discussing a dog’s overweight |
| I am capable of composing an effective treatment plan for overweight |
| I know to whom / which organization I can refer an overweight dog to |
| There is enough information materials available on overweight in dogs for pet owners |
| I create awareness in dog owners regarding the consequences of overweight |
| Possible motivator: Feeling responsible |
| Answer categories: Totally disagree – disagree – neutral – agree – totally agree |
| The vet should tell dog owners when their dog is overweight |
| It's my responsibility to discuss overweight |
| It is my responsibility to compose an effective treatment plan for overweight |
| It is my responsibility to create awareness in dog owners regarding the consequences of overweight |
| Possible motivator: Feeling compassion |
| Answer categories: Totally disagree – disagree – neutral – agree – totally agree |
| It makes me feel sad when I see an overweight dog |
| Overweight affects the dog’s welfare |
| Overweight treatment plans affect the dog’s welfare |
| Other |
| Note any other barriers you experience when discussing overweight, which were not addressed during the survey. |
| Any questions or comments can be noted here. |
