# Supplementary material for: Discussing overweight in dogs during a regular consultation in general practice in the Netherlands
Source: J Anim Physiol Anim Nutr (Berl). 2021 Jun 18;105(Suppl 1):56–64. doi: 10.1111/jpn.13558 (PMC8519133; doi:10.1111/jpn.13558)
Supplement: Supplementary file 2 — Appendix 2 [file JPN-105-56-s002.docx]

Appendix 2. Questions for semi-structured face-to-face interviews

| Personal information |
| --- |
| \| Practice name: \|  \| Date: \|  \| Time: \|  \| \| --- \| --- \| --- \| --- \| --- \| --- \| |
| \| Name respondent: \|  \| \| --- \| --- \| |
| \| Practice location: \|  \| Phone number: \|  \| \| --- \| --- \| --- \| --- \| |
|  |
| Questions |

1. General information

- When do you consider a dog overweight?
- What percentage of dogs in your practice is overweight?

1. Discussing overweight (and asking for barriers)

- What is, according to you, the task of a veterinarian regarding overweight in dogs?
- When do you discuss overweight with a dog owner?
- How do you discuss overweight during a consultation not related to overweight?
- What problems do you experience when discussing overweight in dogs?

1. Self-reflection (and motivation)

- What is your personal motivator to discuss overweight?
- What is the strongest barrier you encounter when discussing overweight?
- On what level do you think improvement is possible for yourself when discussing overweight?

1. Knowledge and expertise (skills)

- How do you motivate owners to start a treatment plan for overweight?
- What do you think about the information on overweight available for veterinarians?
- Wat kind of post-graduate education did you follow? (Also on communication skills)
- Do you think you have the knowledge and expertise regarding the subject of overweight? (signaling, discussion, and treatment)
- Can you give me an impression on how you treat overweight dogs?

1. Risk factors

- To what extent, do you think an owner is responsible for a dog’s overweight?
- To what extent, do you think that the social class of the owners is of influence on the overweightness of the dog?
- Are there any other environmental factors that you think are of influence on a dog being overweight?

6. Improvement

- Are there any tips and tricks you apply when discussing overweight? If yes, which one(s)?
- What do you think should change, so that overweight is discussed more often during a consultation that is not related to overweight?
