# Supplementary material for: Discussing overweight in dogs during a regular consultation in general practice in the Netherlands
Source: J Anim Physiol Anim Nutr (Berl). 2021 Jun 18;105(Suppl 1):56–64. doi: 10.1111/jpn.13558 (PMC8519133; doi:10.1111/jpn.13558)
Supplement: Supplementary file 3 — Appendix 3 [file JPN-105-56-s003.docx]

Appendix 3. Comments section online survey

|  |  |
| --- | --- |
| 3 | Sometimes it is difficult if owners simply do not want to see the problem and the consequences, and they keep telling you that their dog is not overweight. |
| 4 | Overweight colleagues will be pointed out on their own weight when trying to discuss overweight. |
| 5 | It is difficult addressing overweight with someone who doesn’t acknowledge the problem. Some people simply do not know and are eager for help, others do not see the problem. |
| 7 | Compliance and follow up. Weighing their dog on a regular basis. The information seems to be quickly forgotten by the owner. |
| 10 | Opinions from neighbors, friends, and acquaintances often do not match my opinion about the dog being overweight. The owner finds it hard to acknowledge our recommendations and starts doubting the severity of the dog’s overweight. Many people find dogs with a good BCS too thin. The existence of an obstinately distorted image of how a healthy dog (and cat) looks like. |
| 12 | Some owners just don’t care. |
| 13 | People who do not agree with me that their dog is overweight/obese. |
| 17 | A lot of owners are convinced that their dog does not eat a lot of food, I find it hard to explain this to them. Some owners just do not want to hear it. |
| 19 | There are more important problems than overweight animals. |
| 24 | Indifference of the owners, or unwillingness. |
| 27 | Overweight owners. Owners that start to defend themselves as soon as the topic is brought up. |
| 28 | I sometimes find it hard to discuss overweight with overweight owners, but I do discuss it. Difficulty occurs with defensive owners or if they blame someone else. |
| 31 | Lack of time during consultation, sometimes due to other or more severe problems. |
| 33 | Lack of time to explain the severity of the problem. Also, explaining co-morbidities is difficult for me. |
| 33 | More materials about explaining the severity of overweight would be handy. |
| 34 | Owners immediately cut off the conversation and start talking about another topic when I try to address overweight. I do not want to insist then, and make people feel uncomfortable when they leave. |
| 37 | Unwilling owners that do not see the problem. |
| 38 | I am a referral veterinarian, it is difficult to help clients when their own veterinarian had never appointed it. |
| 39 | Lack of motivation (from the owner) |
| 41 | The veterinarian himself is overweight, which makes it harder to appoint owners on their pet’s overweight. |
| 42 | Owners that are not willing to start the weight management program, or owners that do not want a follow up consultation for initiating a treatment plan. |
| 43 | Owners that do not want to hear that their pet is overweight, or owners that are annoyed because they hear the same story every consultation. |
| 44 | Owners that do not recognize the severity of the issue. |
| 54 | Sometimes it is hard to design a treatment plan. Only by telling the owner to feed less often does not work. |
| 59 | Owners that find it too difficult to give their dog less food. |

Green = Resistant owner Red = Indifference of the owner
